# O.2.3-7 Physical activity levels and sociodemographic factors associated with meeting recommended levels among shop attendants in Mbarara municipality, Uganda

**DOI:** 10.1093/eurpub/ckad133.133

**Published:** 2023-09-11

**Authors:** Evas Nimusiima, Solomon Wafula, Hilbert Mendoza, Rawlance Ndejjo, Edwinah Atusingwize

**Affiliations:** Department of Disease Control and Environmental Health, School of Public Health, College of Health Sciences, Makerere University, P.O Box 7072 Kampala, Uganda; Department of Disease Control and Environmental Health, School of Public Health, College of Health Sciences, Makerere University, P.O Box 7072 Kampala, Uganda; Department of Community Health and Behavioural Sciences, School of Public Health, College of Health Sciences, Makerere University, P.O Box 7072 Kampala, Uganda; enimusiima@must.ac.ug; Department of Disease Control and Environmental Health, School of Public Health, College of Health Sciences, Makerere University, P.O Box 7072 Kampala, Uganda; Department of Disease Control and Environmental Health, School of Public Health, College of Health Sciences, Makerere University, P.O Box 7072 Kampala, Uganda

## Abstract

**Purpose:**

Shop attendants are urban dwellers who may spend significant periods in sedentary lifestyles exposing them to non-communicable diseases. This study assessed the physical activity levels and sociodemographic factors associated with meeting the WHO recommended physical activity levels among shop attendants in Mbarara municipality, Uganda.

**Methods:**

We conducted a cross-sectional study among 301 shop attendants. We used the global physical activity questionnaire to assess participants' physical activity levels. Modified Poisson regression was used to assess the sociodemographic factors associated with meeting recommended physical activity levels.

**Results:**

Of the 301 participants, 234 (77.7%) met the WHO physical activity recommendations, especially through work-related physical activity of moderate intensity 194 (64.5%). The median weekly duration of all moderate-intensity physical activity was 180 min (IQR=90 to 360). The median daily sedentary time was 300 min (IQR=300 to 360). Being male (adjusted prevalence ratio=1.33, 95% CI 1.17 to 1.51) was significantly associated with meeting recommended physical activity levels.

**Conclusion:**

The physical activity levels among shop attendants were high and were mostly achieved through work-related activities of moderate intensity, with males being more likely to meet recommended physical activity levels. Findings suggest a need for gender-sensitive initiatives to increase physical activity levels, especially among female shop attendants.

